# Computer-Based Immunoinformatic Analysis to Predict Candidate T-Cell Epitopes for SARS-CoV-2 Vaccine Design

**DOI:** 10.3389/fimmu.2022.847617

**Published:** 2022-03-30

**Authors:** Xueyin Mei, Pan Gu, Chuanlai Shen, Xue Lin, Jian Li

**Affiliations:** ^1^ Key Laboratory of Developmental Genes and Human Disease, Ministry of Education, School of Life Science and Technology, Southeast University, Nanjing, China; ^2^ Department of Math and Computer Sciences, College of Letters and Science, University of Wisconsin–Madison, Madison, WI, United States; ^3^ Department of Microbiology and Immunology, Medical School of Southeast University, Nanjing, China; ^4^ Department of Bioinformatics, School of Biomedical Engineering and Informatics, Nanjing Medical University, Nanjing, China

**Keywords:** SARS-CoV-2, S protein, T-cell epitopes, molecular docking, vaccine

## Abstract

Since the first outbreak of coronavirus disease 2019 (COVID-19), caused by severe acute respiratory syndrome coronavirus 2 (SARS-CoV-2) in 2019, its high infectivity led to its prevalence around the world in an exceptionally short time. Efforts have been made to control the ongoing outbreak, and among them, vaccine developments are going on high priority. New clinical trials add to growing evidence that vaccines from many countries were highly effective at preventing SARS-CoV-2 virus infection. One of them is B cell-based vaccines, which were common during a pandemic. However, neutralizing antibody therapy becomes less effective when viruses mutate. In order to tackle the problem, we focused on T-cell immune mechanism. In this study, the mutated strains of the virus were selected globally from India (B.1.617.1 and B.1.617.2), United Kingdom (B.1.1.7), South Africa (B.1.351), and Brazil (P.1), and the overlapping peptides were collected based on mutation sites of S-protein. After that, residue scanning was used to predict the affinity between overlapping peptide and HLA-A*11:01, the most frequent human leukocyte antigen (HLA) allele among the Chinese population. Then, the binding free energy was evaluated with molecular docking to further verify the affinity changes after the mutations happen in the virus genomes. The affinity test results of three epitopes on spike protein from experimental validation were consistent with our predicted results, thereby supporting the inclusion of the epitope _374_FSTFKCYGL_382_ in future vaccine design and providing a useful reference route to improve vaccine development.

## Introduction

Coronavirus disease 2019 (COVID-19) caused by severe acute respiratory syndrome coronavirus 2 (SARS-CoV-2) has been and is still a large threat to human health. Its name comes from its crown-like spike protein structures. Although SARS-CoV-2 is found to share similar structures with other coronaviruses like SARS-CoV, its higher binding affinity to host cells makes it more transmissible than others and more difficult to control. Its influence has spread to over 200 countries, and more than two billion people have suffered under its infection (1). Vaccines are proven to be one of the most effective ways to prevent the disease and help the world to recover. Around the world, more than 200 vaccine candidates are proposed (2). Besides traditional inactivated virus vaccines, forms of viral vector and subunit vaccine are emerging to increase protectiveness against the virus (1). Although some laudable effects have been achieved, other challenges, including the decreasing effectiveness of circulating vaccines against certain mutated strains of the virus and failure of long-term strong immunity, remain controversial (1). SARS-CoV-2 virus is composed of RNAs, and during its infection of cells, copying errors occur, which are called mutations (3). A group of viruses that possesses similar inherited traits is named as a variant. Due to their differences from the original virus, both prevention and treatment become challenging.

Four structural proteins, spike, membrane, envelope, and nucleocapsid, together form the SARS-CoV-2. Although studies have shown that T-cell epitopes on other proteins can also activate immune response and they can also be useful in vaccine development, compared to spike proteins, they are not as promising as spike proteins in many ways (4). SARS-CoV-2 binds with angiotensin-converting enzyme 2 (ACE2), and its spike (S) protein on the surface of virion mediates virus entry to host cells that is achieved by fusing viral and cellular membrane (1). S protein can be cleaved into S1 part and S2 part. S1 domain is responsible for receptor binding, and S2 protein participates in protein fusion (5). Part of S1 domain functioned as a receptor-binding domain (RBD) so when SARS-CoV-2 virus attacks target cells, such subdomain will bind to ACE2. Then, S2 domain can further process the binding (6). Therefore, mutations on S protein may change binding abilities of the virus and ACE2 and make people more vulnerable to infection. Moreover, study results revealed that over 1,800 mutations occurred on S protein of the virus, and some of the main mutations are a result of S protein mutations. Fortunately, by analyzing every mutation on the S protein, potential epitopes can be discovered and the importance of S protein in virus interaction with human cells makes it attractive to be used as a vaccine target (7).

**Figure 1:**
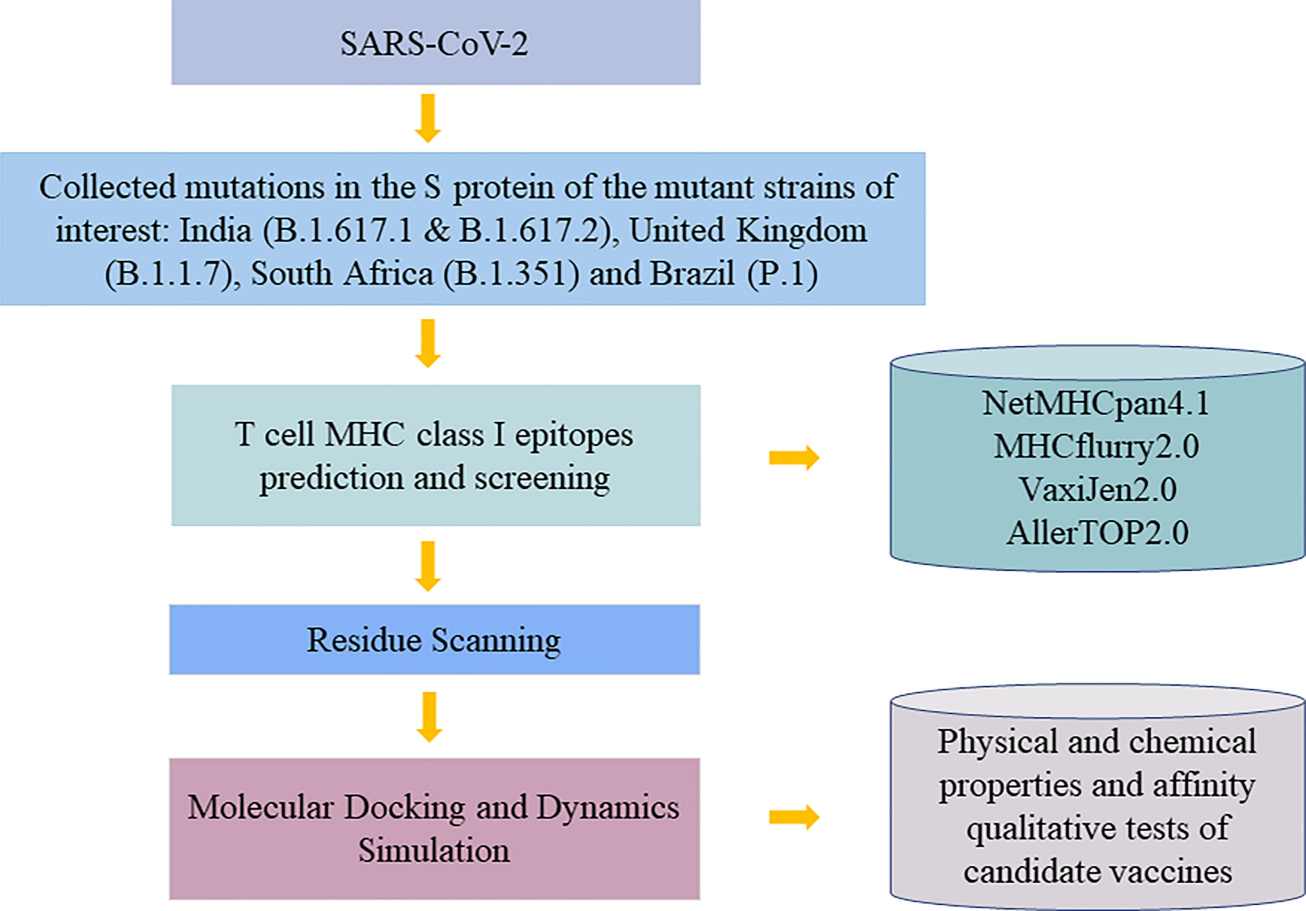
Flowchart in the vaccine design study.

T cells are found to have high correlations with providing immunity against SARS-CoV-2 virus. By targeting T cells, vaccines can trigger both acquired and innate immunity in human bodies (8). Human leukocyte antigen (HLA), as an integral part of viral antigen presentation pathway, plays a crucial role in the occurrence and development of infectious diseases. Virus-specific CD8+ T cells recognize HLA class I peptide complexes and induce apoptosis of infected cells to control viral infection. Recent studies have shown that individual HLA genotypes may differ in inducing T cell-mediated antiviral responses, with HLA-A*11:01 having a relatively high ability to present SARS-CoV-2 antigen. And individuals with HLA-1*11:01 genotype may trigger a more potent T cell-mediated antiviral response to SARS-CoV-2 (9). Therefore, our study aims to discover epitopes with potential to interact with HLA-A*11:01 commonly found in the Chinese population for vaccines to induce long-term immunity. In order to activate CD8+ T cells, potential T-cell epitopes, part of proteins that is in charge of such activation of human immune systems to fight against pathogens is needed to be discovered to be used in epitope-based vaccine designs (10). Traditionally, biology and chemistry experiments were used to find out the epitopes, but due to the high cost of experiments and advancement of technology, mass deployment of bioinformatic tools to provide reliable results and before using experiment as verification greatly lowers the cost and saves time of prediction (1). We started by comparing the S protein amino acid sequence of the original strain with that of variants, and with the help of proto-peptide overlapping peptide library, overlapping peptides covering the mutation site are generated for the next step. We filtered out the peptides with immunogenicity by using NetMHCpan 4.1 (https://www.cbs.dtu.dk/services/NetMHCpan/) to evaluate the affinity between these overlapping peptide and major histocompatibility complex (MHC) molecules and picked HLA peptides with matching amino acids and overlapping peptides with immunogenicity. Binding affinity after mutation between them is predicted by molecular mechanics-generalized Born surface area (MM-GBSA) residue scanning functionality of BioLuminate (version 1.0, Schrödinger, LLC, NY, USA, 2020-1), and pairs with a higher possibility to bind will be recorded. Additionally, molecular docking and dynamics simulation were used to verify the interaction result between protein and peptide. Finally, immunogenic peptide fragments were obtained by qualitative affinity test and physical and chemical properties of the vaccine (**Figure 1**). In this study, the prediction and screening of T-cell epitopes based on computer methods have opened up new doors for the design of effective vaccines.

## Materials and Methods

### Extracting Data

Mutated strains with a high prevalence in the world are picked from India (B.1.617.1 and B.1.617.2), United Kingdom (B.1.1.7), South Africa (B.1.351), and Brazil (P.1). S protein genome sequences of these strains (Accession no. YP_0097243901.1) are downloaded from NCBI database (www.ncbi.nlm.nih.gov) since S protein is responsible for helping the virus enter host cells by their interactions with ACE2 (11). With help from GISAID (www.gisaid.org) and outbreak.info (https://outbreak.info/), we collected a total of 36 mutation sites in the S region of these epidemic variants.

### Filtering Data

The receptor-binding domain (RBD) of SARS-CoV-2 spike protein plays a crucial role in binding to human ACE2, so our focus was on mutations in RBD regain (319-541AA). Side chains of amino acids can be classified as polar or non-polar and hydrophobic or hydrophilic, and because non-polar or weakly polar chains have difficulty interacting with water, they are hydrophobic. Studies have shown that there are many hydrophobic amino acids on the site of antigen and antibody binding (12), so we also took the sites that are mutated into hydrophobic amino acids in the S protein as the research objects of interest. We hypothesized that epitopes generated based on these mutated sites as well as the mutated sites in the most immunogenic region of the S protein named RBD (13) might be more likely to be presented to the cell surface by MHC molecules and recognized by T cells. In addition, the high-frequency D614G mutation that appeared in these five variants was also considered to be interesting, since several studies emphasized the impact of D614G variant on transmission effectiveness (14). Therefore, a total of 18 mutations based on 16 sites were used for proto-peptide, resulting in a 19-length overlapping peptide containing the mutated site.

### T-Cell Major Histocompatibility Complex Class I Epitope Prediction and Screening

In this study, HLA-A*11:01, the most common allele of HLA-I in the Chinese population, was selected for epitope prediction (15). MHC Class I restrictive CD8+ T cell-binding epitopes were identified by using NetMHCpan4.1 (https://services.healthtech.dtu.dk/). The peptide length was 8–14 mer, and the default thresholds of 0.5% strong binding agent and 2% weak binding agent were used to filter out peptide allele with binding affinity. To verify the result and increase accuracy, we also made use of MHCflurry2.0 tool (16) to predict the most possible MHC–peptide binding pairs. Thereafter, VaxiJen2.0 server (http://www.ddg-pharmfac.net/vaxijen/VaxiJen/VaxiJen.html) and AllerTOP v. 2.0 server (https://www.ddg-pharmfac.net/AllerTOP/) were used to screen candidate peptide epitopes with antigenicity and non-allergenicity.

### Residue Scanning With Prime Molecular Mechanics-Generalized Born Surface Area

Human MHC-I molecules are highly polymorphic and have a specific peptide motif preference (17). In order to determine the HLA structure to which the immunogenic peptide is attached, we used residue scanning module with MM-GBSA method in Schrödinger software (LLC, NY, USA, 2020-1) to select structure with the strongest affinity as the subsequent docking receptor. The HLA 3D structure of this research was downloaded from Protein Data Bank (RCSB PDB) (https://www.rcsb.org/). According to binding properties of HLA polypeptides where the second position and the end position are significant binding sites, the HLA structure with high consistency with the amino acids in the binding pocket of the predicted epitope peptide was preferentially chosen to get mutation.

Binding affinity after mutation between immunogenic peptides and HLA-derived peptides is calculated by MM-GBSA Residue Scanning functionality of BioLuminate (version1.0, Schrödinger, LLC, NY, USA, 2020-1). Concurrent mutations of all amino acids are allowed, and side-chain prediction with backbone sampling refinement is chosen. Although according to Schrödinger’s official guide, the affinity calculated from the tool may not be consistent with the experimental result, the ranking of affinity result agrees with the experimental result. Therefore, a lower binding affinity represents stronger binding. Some studies used 3 kcal/mol to define places where mutations may have a strong influence on affinity (18). Here, we use 5 kcal/mol to define the boundary. Thus, after calculating affinity before and after mutations, mutations with an increase in affinity and value not greater than 5 kcal/mol are selected for the next step.

### Molecular Docking

Glide (Schrödinger 2020-1 release) uses a series of hierarchical filters to search for possible favorable peptide interactions at receptor-binding sites (19). In molecular docking study, preprocessed HLA3D structure was used as the starting structure in Peptide Docking of Glide module, and the peptide poses were generated by inputting the sequence of epitopes. In the docking process, the binding sites of HLA peptides need to be defined, and the network box was generated to define the docking location. Using the Superposition workbench, the pose with the highest backbone similarity [lowest root mean square deviation (RMSD) value] with the original skeleton will be used for the next step.

### Molecular Dynamics Simulation

For molecular dynamics (MD) simulation, maestro’s Desmond module (Schrödinger 2020-1 release) was used. A system builder panel with the OPLS3e force field was used to set up a biological system before MD simulation. The SPC model of water considered for solvating the system and an orthorhombic box with a 10Å buffer distance was generated. In this work, a total of 100-ns MD simulation was running on a GPU at 300K temperature and 1.01325 bar pressure. Simulation Interaction Diagram shows the interaction of peptide–protein such as RMSD images that determine the stability of the complex. The simulated complex was visualized by Pymol software (version 2.0, Schrödinger, LLC) to observe the polar contacts between ligand and receptor.

### Experimental Validation of Affinity Between Candidate Peptides and HLA-A*11:01

The Octet^®^ system is based on Bio-Layer Interferometry (BLI) to screen and characterize molecular interactions (20). The binding affinities of HLA-A*11:01 protein and candidate epitopes were performed on the Octet R8 system (Sartorius, German). Biotinylated protein was immobilized on SSA sensors for 20 min, and the signal approached a height of about 4 nm. PBST buffer solution [phosphate buffered saline (PBS)+0.02%Tween20+1% dimethyl sulfoxide (DMSO)] was utilized to dilute the peptides to 100, 50, 25, 12.5, 6.25, 3.125 µM, respectively. The whole polypeptide–protein interaction system was balanced, bound, and dissociated for 60 s, and the binding curve was drawn using Octet^®^ analysis studio 12.2 software. The final epitopes were concluded from analyzing the physicochemical properties of the candidate epitopes obtained by ProtParam online tool of ExPASy server (https://web.expasy.org/protparam/) and the experimental results of affinity.

## Results

### Amino Acid Mutation in S Region of Five Mutants

**Figure 2:**
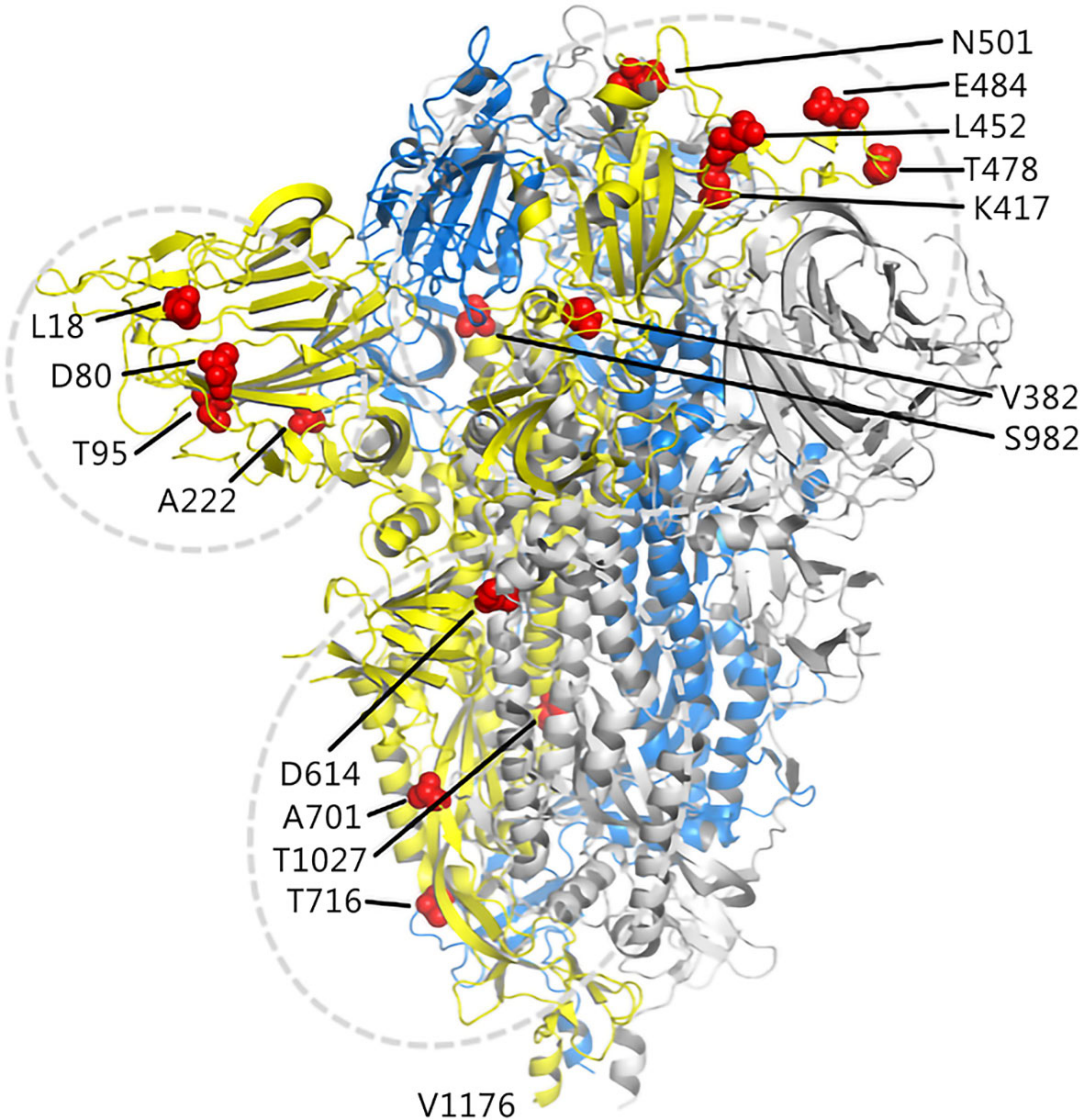
Illustration of candidate sites on the S protein. The three chains of the trimer structure of S protein (PDB 7DF3) are shown in cartoon and represented as yellow, marine, and gray. The mutation sites of interest are shown as red spheres.

In this study, details about 16 mutation sites of interest on S protein obtained from five SARS-COV-2 variants are shown in **Figure 2**.

### T-Cell Epitope Prediction and Analysis of Antigenicity and Hypersensitivity

A total of 38 potential T-cell epitopes were predicted by the NetMHCpan4.0 server and MHCflurry, which docked on the HLA-A*11:01 allele in an energy-favorable manner. Antigenicity was predicted using the VaxiJen tool, with a threshold of 0.4 for possible antigens (21). **Table 1** shows the antigenic and non-allergenic epitopes paired with HLA-A*11:01 after screening, and their VaxiJen scores resulted in the optimal 13 epitopes based on the 7 mutation sites.

**Table 1:** T cell-predicted epitopes of selected mutation sites based on antigenicity and allergenicity.

MHC I Allele	Mutation Sites	Epitopes	VaxiJen Score	Antigenicity	Allergenicity
HLA-A*11:01	L18F	SQCVNFTTR	1.7440	Probable antigen	Non-allergen
		SSQCVNFTTR	1.7772	Probable antigen	Non-allergen
	T95I	GVYFASIEK	0.4008	Probable antigen	Non-allergen
	V382L	KCYGLSPTK	1.6366	Probable antigen	Non-allergen
		GLSPTKLNDL	1.7584	Probable antigen	Non-allergen
		FSTFKCYGL	0.5941	Probable antigen	Non-allergen
	K417T	GTIADYNYK	1.8607	Probable antigen	Non-allergen
		TGTIADYNYK	1.7029	Probable antigen	Non-allergen
	L452R	KVGGNYNYR	1.5212	Probable antigen	Non-allergen
	T1027I	ASANLAAIK	0.5724	Probable antigen	Non-allergen
	V1176F	GINASFVNIQK	0.9624	Probable antigen	Non-allergen
		INASFVNIQK	1.1168	Probable antigen	Non-allergen
		NASFVNIQK	1.2242	Probable antigen	Non-allergen

These red fonts represent mutated amino acids and are presented as single-letter abbreviations.

### Residue Scanning of Predicted CD8 T-Cell Epitopes With HLA-A*11:01

The anchor position of HLA self-binding peptides is mainly the second and terminal amino acids. Considering the properties and lengths of amino acids of the candidate epitopes, we selected the structure with the most similar peptide docking pattern from HLA-A*11:01 for amino acid mutation prediction. In this study, 57 affinity values that were obtained from these 13 epitopes are shown in **Table S1**. Here, we show the performance of each candidate epitope with HLA-A*11:01 receptor after initial screening (**Table 2**). Since a smaller affinity represents greater binding abilities of mutant bond than its parent protein, we filtered candidate epitopes with a threshold of △affinity of 5 kcal/mol (22) for further kinetic studies. During the analysis, _13_SQCVNFTTR_21_, which exhibited strong affinity with a change value of -10.06 kcal/mol, and peptide _378_KCYGLSPTK_386_, with △affinity value of 4.46 kcal/mol, were both included in the subsequent study. Inherent in this approach is the problem that some HLA structures like 5GRD cannot be directly mutated because two cysteines will form disulfide bonds, and candidate peptides are inconsistent with HLA self-binding peptides in number. To circumvent this, we directly performed molecular docking in Glide module of Schrödinger software (LLC, NY, USA, 2020-1) on these structures.

**Table 2:** The performance of selected epitopes and HLA structures screened according to the minimum △affinity.

Mutation Sites	Epitopes	HLA-A*11:01 PDB ID	△ Affinity kcal/mol	△ Stability kcal/mol	△ Hydropathy	△ Prime Energy kcal/mol
L18F	SQCVNFTTR	1Q94	-10.06	15.78	1.59	-59.45
	SSQCVNFTTR	5GRD	Docking			
		2HN7	-1.46	40.9	2.46	-108.71
T95I	GVYFASIEK	1Q94	12.28	-4.83	1.31	28.19
V382L	KCYGLSPTK	1X7Q	4.46	6.74	1.98	-34.89
	GLSPTKLNDL	5GRD	Docking			
		2HN7	46.13	21.03	-0.10	-18.60
	FSTFKCYGL	5GRD	Docking			
		1Q94	48.44	28.29	0.82	105.10
K417T	GTIADYNYK	1Q94	24.29	16.60	-0.02	-9.39
	TGTIADYNYK	1QVO	45.73	4.00	-0.53	-32.80
L452R	KVGGNYNYR	1QVO	Docking			
		1X7Q	8.70	8.65	-0.74	-159.55
T1027I	ASANLAAIK	6JOZ	37.35	-0.85	0.19	14.91
V1176F	GINASFVNIQK	4MJ5	51.90	45.37	-1.86	7.21
	INASFVNIQK	1QVO	31.29	2.31	0.74	-53.95
	NASFVNIQK	6JOZ	27.27	5.48	1.55	-68.51

These red fonts represent mutated amino acids and are presented as single-letter abbreviations.

### Molecular Dynamics Simulation of the Post-Docking Structure

Peptide Docking in the Biologics module of Schrödinger software was used to study the interaction between HLA molecules and epitope peptides. The Docking result was reflected by the RMSD value of carbon skeleton. A smaller RMSD value indicates higher similarity in the structure of the docking peptide is with the conformation of the original HLA peptide (23), which allows for optimal matching of shapes and interactions between epitopes and HLA molecules. Following the screening after residue scanning calculation, seven epitopes and their wild-type peptides were docked with the corresponding HLA receptor, and the docked model was used as the initial structure of dynamics simulation.

In this work, Desmond module (Schrödinger 2020-1 release) was used to conduct 100-ns simulation, and RMSD values of the system provided a good estimate of whether the docking complex was in a stable state (24). Our study found that the RMSD values of the complexes of HLA-A*11:01 and the epitopes based on different mutation sites were different from those of the wild-type complexes. These complexes reach their own stable conformation at different times, and their stability might fluctuate slightly over time.

**Figure 3:**
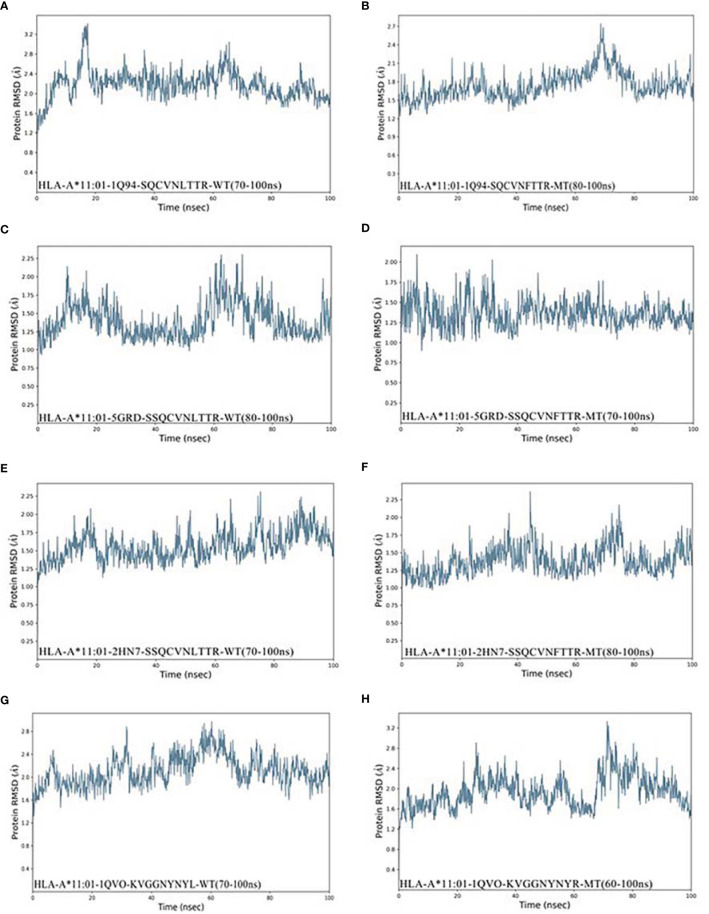
RMSD plot of the HLA-A*11:01-epitopes, indicating stability. **(A)** HLA-A*11:01(1Q94)-SQCVNLTTR (WT). **(B)** HLA-A*11:01(1Q94)-SQCVNFTTR (MT). **(C)** HLA-A*11:01(5GRD)-SQCVNLTTR (WT). **(D)** HLA-A*11:01(5GRD)-SQCVNFTTR (MT). **(E)** HLA-A*11:01(2HN7)-SSQCVNLTTR (WT). **(F)** HLA-A*11:01(2HN7)-SSQCVNFTTR (MT). **(G)** HLA-A*11:01(1QVO)-KVGGNYNYL (WT). **(H)** HLA-A*11:01(1QVO)-KVGGNYNYR (MT). RMSD, root mean square deviation; MT, mutation type; WT, wild type.

It was observed from **Figures 3A, B** that the stable conformation of _13_SQCVNFTTR_21_-HLA-A*11:01(1Q94) complex was achieved at RMSD 1.7 Å. However, in the dynamic system before this epitope mutation, there is still a downward fluctuation until the last 50 ns. In the case of the RMSD of peptide _12_SSQCVNFTTR_21_ compound with HLA-A*11:01 (5GRD), the interaction was observed to become stable and at ~1.25 Å in the end. Differently, the results of RMSD of _12_SSQCVNLTTR_21_ complex displayed a substantial fluctuation between 0 and 100 ns. As for another HLA-A*11:01 crystal structure 2HN7 docking with _12_SSQCVNFTTR_21_ epitope, the RMSD value of the complex showed a slight upward trend. We can also find it was unstable before the mutation (**Figures 3C–F**). In addition, **Figures 3G, H** represents that _444_KVGGNYNYR_452_-HLA-A*11:01(1QVO) complex has a large fluctuation. While assessing the RMSD of wild compound _444_KVGGNYNYL_452_-HLA-A*11:01(1QVO), the results of RMSD displayed a clear upward trend between 0 and 60 ns, but subsequently, the compound got stable.

**Figure 4:**
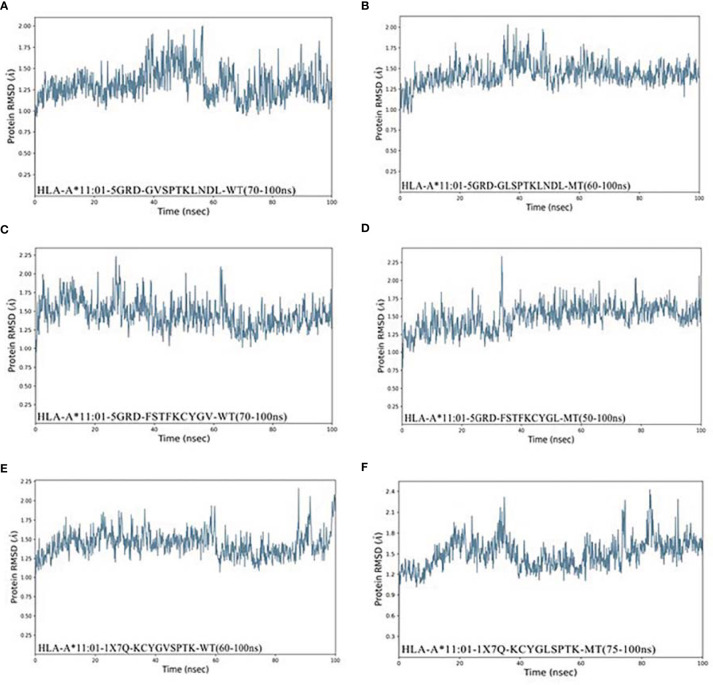
RMSD plot of the HLA-A*11:01-epitopes based on V382L. **(A)** HLA-A*11:01(5GRD)-GVSPTKLNDL (WT). **(B)** HLA-A*11:01(5GRD)-GLSPTKLNDL (MT). **(C)** HLA-A*11:01(5GRD)-FSTFKCYGV (WT). **(D)** HLA-A*11:01(5GRD)-FSTFKCYGL (MT). **(E)** HLA-A*11:01(1X7Q)-KCYGVSPTK (WT). **(F)** HLA-A*11:01(1X7Q)-KCYGLSPTK (MT). RMSD, root mean square deviation; MT, mutation type; WT, wild type.

As shown in **Figures 4A–F**, Compound HLA-A*11:01 (5GRD) and _381_GLSPTKLNDL_390_ attained 1.25Å RMSD at the beginning of simulation and remained stable between 1.25 and 1.5Å after 60 ns, while its wild system fluctuates slightly in the last 50 ns. In the case of _374_FSTFKCYGL_382_-HLA-A*11:01 (5GRD) complex, it was observed to have a steady increase from 0 to 50 ns in its RMSD result but subsequently stabilized between 1.25 and 2.0 Å after 50 ns, and its wild system is relatively balanced. Notably, the RMSD trajectories of complex consisting of _378_KCYGLSPTK_386_ exhibited a significant fluctuation in binding to the receptor of HLA-A*11:01(1X7Q). The complex reached ~2.4 Å from 0 to 35 ns; however, this value remarkably decreased after 35 ns and remained stable at 40~70 ns and finally stabilized around 1.6 Å after an upward fluctuation. Differently, the RMSD of its wild peptide _378_KCYGVSPTK_386_ compound with HLA-A*11:01(1X7Q) remained stable in the early stage, but fluctuated in the latter 20 ns.

### MM-GBSA Calculates the Binding Free Energy

In order to determine the binding interaction between candidate epitopes and MHC-I molecules, we quantitatively evaluated the binding free energy of the peptide–HLA complex using MM-GBSA method (25). We obtained the MM-GBSA results of 1,000 frames of complex with 125 frames interval and selected the corresponding frame interval to calculate the average value according to the stable region of RMSD value of the optimized conformation by MD. As predicted in **Table 3**, our results demonstrate the changes in binding free energy of site-based epitopes before and after mutation. Except for epitopes _12_SSQCVNFTTR_21_ and _444_KVGGNYNYR_452_, the remaining mutation-based epitopes showed more favorable binding free energy than wild-type peptide, that is, more negative numerically. It is noteworthy that three candidate epitopes obtained by mutation V382L all showed favorable binding to HLA-A*11:01 after mutation. Among them, _374_FSTFKCYGL_382_ achieved the strongest binding free energy (-91.949 kcal/mol) after mutation among all candidate epitopes. In other words, these four peptides (_13_SQCVNFTTR_21_/_378_KCYGLSPTK_386_/_381_GLSPTKLNDL_390_/_374_FSTFKCYGL_382_) are more likely to bind to T cells.

**Table 3:** Results of binding free energy after MD simulation with HLA-A*11:01 and selected epitopes.

Mutation Sites	Epitopes	HLA-A*11:01	Binding Free Energy	Binding Free Energy
		PDB ID	(WT) kcal/mol	(MT) kcal/mol
L18F	SQCVNFTTR	1Q94	-40.506	-78.177
	SSQCVNFTTR	5GRD	-88.204	-51.707
	SSQCVNFTTR	2HN7	-68.863	-57.243
V382L	KCYGLSPTK	1X7Q	-60.699	-77.538
	GLSPTKLNDL	5GRD	-54.390	-85.368
	FSTFKCYGL	5GRD	-36.461	-91.949
L452R	KVGGNYNYR	1QVO	-93.064	-49.170

These red fonts represent mutated amino acids and are presented as single-letter abbreviations.

### Interaction Analysis of Candidate Epitopes

**Figure 5:**
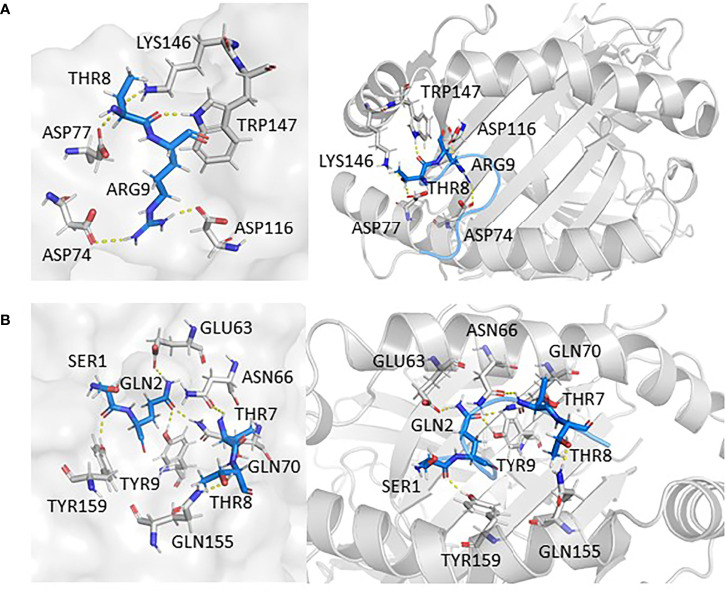
The interaction between epitopes obtained from L18F and HLA-A*11:01. Here, epitopes are shown in marine, HLA structure is shown in gray. **(A)** HLA-A*11:01(1Q94)-SQCVNLTTR (WT). **(B)** HLA-A*11:01(1Q94)-SQCVNFTTR (MT). RMSD, root mean square deviation; MT, mutation type; WT, wild type.

In the study, epitopes based on mutation L18F and V382L showed potential binding against HLA-A*11:01. In the case of L18F, epitope _13_SQCVNFTTR_21_ established six polar contacts with TYR9, GLU63, ASN66, GLN70, GLN155, and TYR159 of HLA-A*11:01(1Q94), while the wild one possessed one less interaction with residues ASP74, ASP77, ASP116, LYS146, and TRP147. From the perspective of the configuration of peptide binding to HLA, the candidate epitopes are more consistent with the conformation of peptide docking with HLA, which is convex in the middle and flat at both ends (**Figures 5A, B** **)**.

**Figure 6:**
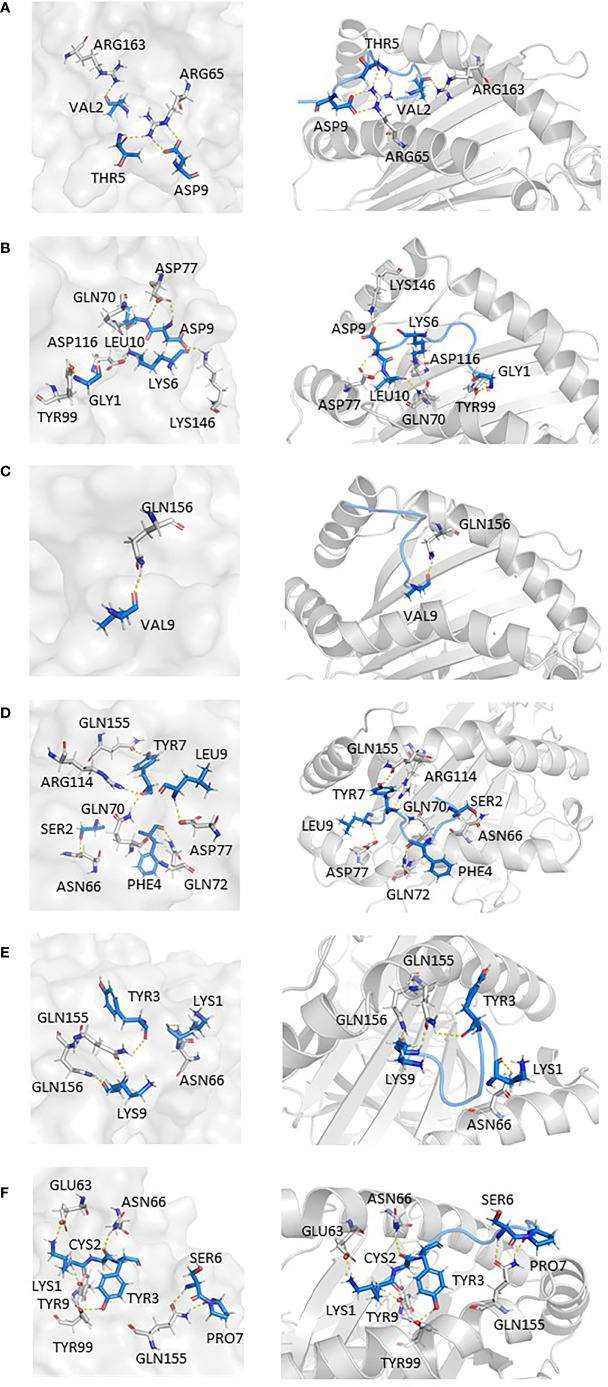
Epitopes obtained from V382L interaction with HLA-A*11:01; epitopes are shown in marine, HLA structure is shown in gray. **(A)** HLA-A*11:01(5GRD)-GVSPTKLNDL (WT). **(B)** HLA-A*11:01(5GRD)-GLSPTKLNDL (MT). **(C)** HLA-A*11:01(5GRD)-FSTFKCYGV (WT). **(D)** HLA-A*11:01(5GRD)-FSTFKCYGL (MT). **(E)** HLA-A*11:01(1X7Q)-KCYGVSPTK (WT) **(F)** HLA-A*11:01(1X7Q)-KCYGLSPTK (MT).

In addition, findings within **Figures 6A–F** presented the interaction information of other epitopes from V382L. _381_GLSPTKLNDL_390_–HLA-A*11:01(5GRD) complex was stabilized by six polar contacts with amino acid residues GLN70, ASP77, TYR99, ASP116, and LYS146, while the wild one formed four bond interactions with residues ARG65 and ARG163 (**Figures 6A, B** **)**. As for _374_FSTFKCYGL_382_, it possessed six polar contacts with residues ASN66, GLN70, GLN72, ASP77, ARG114, and GLN155 of HLA-A*11:01(5GRD), whereas its wild-type _374_FSTFKCYGV_382_ only had one interaction with residue GLN156 (**Figures 6C, D** **)**. Another epitope peptide _378_KCYGLSPTK_386_ was also obtained based on V382L; it expressed six interactions with residues TYR9, GLU63, ASN66, TYR99, and GLN155 of HLA-A*11:01(1X7Q). By contrast, its wild type formed only four interactions (**Figures 6E, F** **)**.

### Physicochemical Properties and Affinity Experiments of Candidate Epitopes

**Figure 7:**
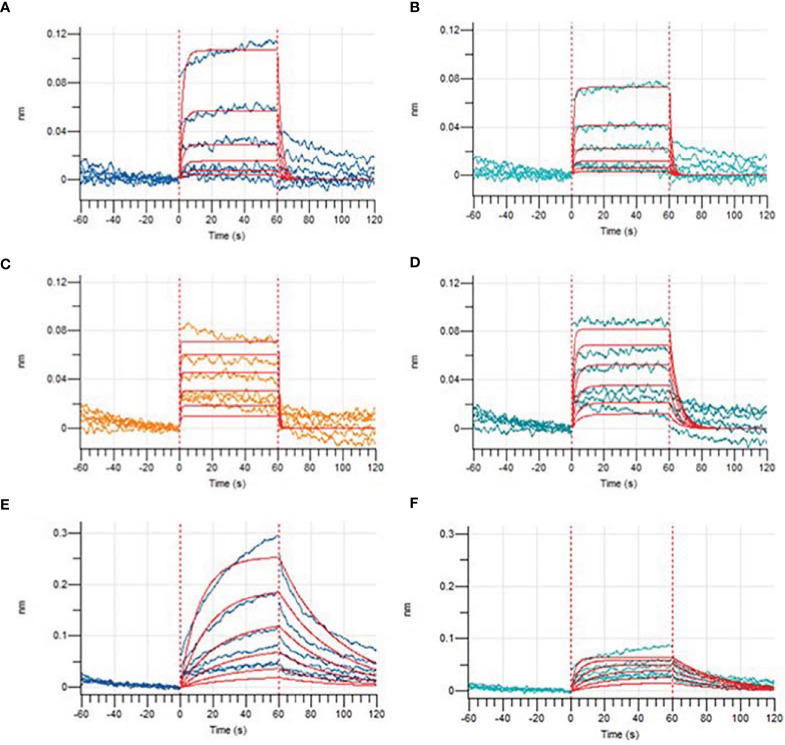
Binding affinities of epitopes before and after mutations obtained by Bio-Layer Interferometry (BLI). Binding dissociation curve between wild-type peptide GVSPTKLNDL **(A)**, KCYGVSPTK **(C)**, FSTFKCYGV **(E)**, mutated epitope GLSPTKLNDL **(B)**, KCYGLSPTK **(D)**, and FSTFKCYGL **(F)** and HLA-A*11:01.

Based on previous computer predictions, higher binding affinities to HLA-A*11:01 that may ultimately affect T-cell clearance were observed in four epitopes, and the results were further confirmed by BLI for assay analysis. After the biotinylated synthetic peptides were immobilized on the super streptavidin (SSA) sensor, the binding and dissociation curves were monitored in real time. As shown in **Figure 7**, dynamic fitting results indicate that the experiment is a typical process of fast combination and fast dissociation. Here we reported the affinities between the epitope _13_SQCVNFTTR_21_ and HLA molecule; its wild-type peptide and HLA molecule were not detected. However, the epitope peptides obtained based on V382L mutation showed relatively ideal affinity values, and the data exhibited a stronger binding between the mutant epitope and HLA molecule. It is worth noting that the epitope _374_FSTFKCYGL_382_ provides a relatively small dissociation constant (KD = 5.947E-06M) through sufficient interaction (**Table 4**). In order to obtain a more comprehensive prediction, we further used ProtParam to calculate multiple physical and chemical parameters of the peptide candidate epitopes and finally screened the epitope _374_FSTFKCYGL_382_. This particular epitope exhibits a theoretical isoelectric point (PI) of 8.20, and the aliphatic index was 43.33 and the instability index was -3.53. The results of physicochemical properties of candidate epitopes are listed in **Table 5**.

**Table 4:** Kinetic analysis by BLI of the binding studies of epitopes to HLA molecular.

Wild Peptide	K_D_ (M)	Epitopes (MT)	K_D_ (M)
SQCVNLTTR	Non-binding	SQCVNFTTR	Non-binding
KCYGVSPTK	2.347E-05	KCYGLSPTK	2.302E-05
GVSPTKLNDL	6.557E-04	GLSPTKLNDL	3.190E-04
FSTFKCYGV	2.911E-05	FSTFKCYGL	5.947E-06

MT, mutation type.

**Table 5:** Physicochemical properties of candidate epitopes.

Candidate Epitopes	Theoretical PI	Instability Index (Indication)	Aliphatic Index	Half-Life (Mammalian reticulocytes)
SQCVNFTTR	7.96	-3.17 (stable)	32.22	1.9 h
KCYGLSPTK	9.20	48.28 (unstable)	43.33	1.3 h
GLSPTKLNDL	5.84	44.45 (unstable)	117.00	30 h
FSTFKCYGL	8.20	-3.53 (stable)	43.33	1.1 h

Theoretical PI, theoretical isoelectric point.

## Discussion

At present, different vaccine strategies are being used in vaccine research for COVID-19, such as RNA, non-replicating viral vectors, peptides, and DNA (26). Among them, peptide vaccines have attracted wide attention due to their antiviral, antitumor, and other infective functions, as well as the advantages of cheapness, safety, and strong specificity. As with natural infections, SARS-CoV-2 vaccination stimulates a strong cellular and humoral immune response that plays a vital protective role in the body (27). Safavi et al. (28) pointed out that T-cell immunity may be more durable than humoral immunity in controlling the novel coronavirus infection. Some other studies have also demonstrated that CD8+ T cells can generally target a variety of SARS-CoV-2 antigens and recognize epitopes from various viral antigens through a series of combinations of T-cell receptors (TCRs), which are crucial for mediating viral clearance and are key to long-term immunity, and protection memory CD8+ T cells can provide against secondary infection (29). Spike protein of SARS-CoV-2 has been included in the focus of vaccine design because of its high specificity and ability to induce a strong immune response. In particular, the RBD region is widely considered as a key protein target for vaccine design and development of neutralizing antibodies as therapeutic agents (30).

In this study, we used immunoinformatics methods to analyze the S protein of SARS-CoV-2 variants prevalent in the world and various advanced tools to identify potential T-cell epitopes of SARS-CoV-2. Here, we conducted further screening of epitopes through residue scanning calculation to obtain antigenicity and non-allergenicity candidate epitopes for molecular docking and dynamics simulation. Our MD simulations revealed that four epitopes (_13_SQCVNFTTR_21_, _378_KCYGLSPTK_386_, _381_GLSPTKLNDL_390_, and _374_FSTFKCYGL_382_) were involved in the interaction with HLA-A*11:01 in a more active way than prior to the mutation. Evidenced by ProtParam’s (31) analysis on physicochemical properties of these selected epitopes, epitopes _378_KCYGLSPTK_386_ and _381_GLSPTKLNDL_390_ with instability index >40 are not considered. To verify results from these four epitopes, we performed wet experiments, in which the affinity between _13_SQCVNFTTR_21_ and HLA-A*11:01 was not detected as expected. Due to satisfactory results in both qualitative affinity experiments and physicochemical analysis, we finally proposed _374_FSTFKCYGL_382_ epitope for the design of vaccine against SARS-CoV-2.

Now, the Omicron variant, which originated in South Africa, has been declared a variant of concern (VOC) by the World Health Organization. Studies have shown that Omicron carries a large number of mutations that may be associated with immune evasion. With the rapid spread of Omicron variants around the world, we also extended our research to Omicron (32). According to the complete study procedure presented in this study, we analyzed the effect of mutations in the S protein of Omicron strain on binding affinity of HLA-A*11:01-restricted CD8+ T-cell epitopes. Seventy epitopes containing mutated sites predicted by NetMHCpan 4.1 and MHCflurry2.0 were obtained. After screening for antigenicity and allergenicity, as well as affinity predicted by residue scanning module, 13 epitopes were finally used for MD simulation. After filtering out the unstable epitopes, we obtained four epitopes worthy of subsequent experimental verification. Our data showed that epitopes _366_SVLYNLAPFFAFK_378_ containing S371L, S373P, S375F, and T376A (binding free energy: -75.434 kcal/mol), _539_VNFNFNGLK_547_ containing T547K (binding free energy: -113.461 kcal/mol), _672_ASYQTQTK_679_ containing N679K mutation sites (binding free energy: -66.8471 kcal/mol), and _672_ASYQTQTKSHR_682_ containing N679K and P681H mutation sites (binding free energy: -106.701 kcal/mol) showed stronger affinity with HLA-A* 11:01 than their wild peptide.

In recent years, computer-based prediction methods have been widely favored in biological research because of their abilities to generate analytical value in a fast and cost-effective manner and thus have also been used in the early stages of vaccine development. Our study provides a very important and reasonable antigen prediction strategy, including analysis of hotspot mutations, prediction of mutant peptides, screening of immunogenic peptides, and structure-based docking simulation, to help us predict potential T-cell epitopes. However, this study is not flawless and it does have limitations. First of all, relatively few HLA types were covered, and they were mainly targeted at the Chinese population. Therefore, the study of T-cell immunity in a variety of HLA types should be considered in the direction of future studies. Secondly, due to the limitations of computer research, inaccuracy of software algorithms may have an impact on the experimental results. Therefore, the comprehensive evaluation of various analytical methods and the verification of wet experiment can better assist vaccine design.

In conclusion, the application of immunoinformatics enabled us to identify four unique immunogenic non-allergic T-cell epitopes from the S protein of the mutant of interest, promising to be associated with HLA-A*11:01 immune response. In the experiment, the epitope _374_FSTFKCYGL_382_ has a stronger affinity. Our results suggest that site-based immunoinformatics analysis procedures may be useful for the development and detection of specific T-cell epitopes for multiple SARS-CoV-2 variants.

## Data Availability Statement

The original contributions presented in the study are included in the article/**Supplementary Material**. Further inquiries can be directed to the corresponding authors.

## Author Contributions

XM and PG collected and analyzed the data and wrote the article. CS designed this study. XL supervised this study and revised the article. JL designed and organized the whole research. All authors contributed to the article and approved the submitted version.

## Funding

This work was supported by the National Natural Science Foundation of China (31871322, 31900473, 82041006), the COVID-19 Emergency Research Fund of Zhejiang University of China (2020XGZX021), and the Fundamental Research Funds for the Central Universities (2242021k10004). The funding bodies were not involved in the study design, data collection, data analysis, and data interpretation.

## Conflict of Interest

The authors declare that the research was conducted in the absence of any commercial or financial relationships that could be construed as a potential conflict of interest.

## Publisher’s Note

All claims expressed in this article are solely those of the authors and do not necessarily represent those of their affiliated organizations, or those of the publisher, the editors and the reviewers. Any product that may be evaluated in this article, or claim that may be made by its manufacturer, is not guaranteed or endorsed by the publisher.
